# Noninvasive estimation of aortic hemodynamics and cardiac contractility using machine learning

**DOI:** 10.1038/s41598-020-72147-8

**Published:** 2020-09-14

**Authors:** Vasiliki Bikia, Theodore G. Papaioannou, Stamatia Pagoulatou, Georgios Rovas, Evangelos Oikonomou, Gerasimos Siasos, Dimitris Tousoulis, Nikolaos Stergiopulos

**Affiliations:** 1 Laboratory of Hemodynamics and Cardiovascular Technology, Swiss Federal Institute of Technology, MED 3.2922, 1015 Lausanne, Switzerland; 2 First Department of Cardiology, Hippokration Hospital, Medical School, National and Kapodistrian University of Athens, Athens, Greece

**Keywords:** Biomedical engineering, Machine learning, Predictive medicine

## Abstract

Cardiac and aortic characteristics are crucial for cardiovascular disease detection. However, noninvasive estimation of aortic hemodynamics and cardiac contractility is still challenging. This paper investigated the potential of estimating aortic systolic pressure (aSBP), cardiac output (CO), and end-systolic elastance (E_es_) from cuff-pressure and pulse wave velocity (PWV) using regression analysis. The importance of incorporating ejection fraction (EF) as additional input for estimating E_es_ was also assessed. The models, including Random Forest, Support Vector Regressor, Ridge, Gradient Boosting, were trained/validated using synthetic data (n = 4,018) from an in-silico model. When cuff-pressure and PWV were used as inputs, the normalized-RMSEs/correlations for aSBP, CO, and E_es_ (best-performing models) were 3.36 ± 0.74%/0.99, 7.60 ± 0.68%/0.96, and 16.96 ± 0.64%/0.37, respectively. Using EF as additional input for estimating E_es_ significantly improved the predictions (7.00 ± 0.78%/0.92). Results showed that the use of noninvasive pressure measurements allows estimating aSBP and CO with acceptable accuracy. In contrast, E_es_ cannot be predicted from pressure signals alone. Addition of the EF information greatly improves the estimated E_es_. Accuracy of the model-derived aSBP compared to in-vivo aSBP (n = 783) was very satisfactory (5.26 ± 2.30%/0.97). Future in-vivo evaluation of CO and E_es_ estimations remains to be conducted. This novel methodology has potential to improve the noninvasive monitoring of aortic hemodynamics and cardiac contractility.

## Introduction

Clinical parameters directly measured in the heart or at the root of the aorta are crucial for detection, diagnosis, prognosis, treatment, and management of cardiovascular diseases^1–4^. Aortic hemodynamics, such as aortic systolic blood pressure (aSBP) and cardiac output (CO), are direct and more informative parameters for assessing cardiovascular health than corresponding measurements obtained at the peripheral arteries^1,5,6^. However, the gold standard techniques for measuring aSBP and CO are catheter-based and expensive^7,8^. Furthermore, there is a need for noninvasive estimation of cardiac contractility. End-systolic elastance (E_es_), i.e., the slope of the end-systolic pressure–volume relation (ESPVR), is a pivotal determinant of left ventricular (LV) systolic performance and a powerful index of the arterio-ventricular interaction^4,9,10^. Despite its clinical importance, the clinical use of this measure is limited by the need for invasive acquisition of multiple LV pressure–volume loops under varying loading conditions^11^.

Peripheral blood pressure (BP) measurements acquired by cuff sphygmomanometry have a fundamental role in the everyday clinical setting^12^. Recognizing the important differences between peripheral and central aortic pressures, significant efforts were oriented towards the noninvasive estimation of aortic hemodynamics, in particular aSBP, based on peripheral pressure measurements^13^. Among commonly used approaches for obtaining aSBP are generalized transfer functions (GTFs)^14–16^, moving average models^17,18^ and pulse wave analysis-based methods^8,19,20^. Nevertheless, the totality of them relies on the acquisition of the entire peripheral pressure waveform which can be tedious and susceptible to errors^21^.

Prediction of CO constitutes a more challenging task due to its dependency on the patient-specific arterial dimensions^22^. Noninvasive CO monitoring has been addressed using single-beat pulse contour analysis^23–25^ which, however, allows for the derivation of only an uncalibrated estimation instead of the absolute CO value. Finally, notable studies have been developed and validated against invasive techniques for estimating E_es_ for a single cardiac cycle^26,27^. The first fully noninvasive method was introduced by Chen et al.^26^. They proposed a simple equation to derive E_es_ from pressure arm-cuffs, echo-Doppler cardiography and electrocardiograms.

Despite the good precision of previous techniques, there has been no holistic and complete study to investigate the possibility of estimating aortic hemodynamics and cardiac contractility using readily available noninvasive measurements on the same population. This is mainly attributed to two inherent limitations, i.e., the lack of invasive data in a large scale and the ethical limitation to perform invasive measurements on a healthy population, if no diagnostic reason has been provided.

Cardiovascular models hold a valuable position for addressing the challenge of limited access on in-vivo data^28,29^. They constitute a faithful representation of the real cardiovasculature and allow the study of pathophysiological mechanisms and diseases^30,31^. Furthermore, they can provide a complete set of parameters to describe the system, whereas the simulated signals are noise-free.

**Figure 1:**
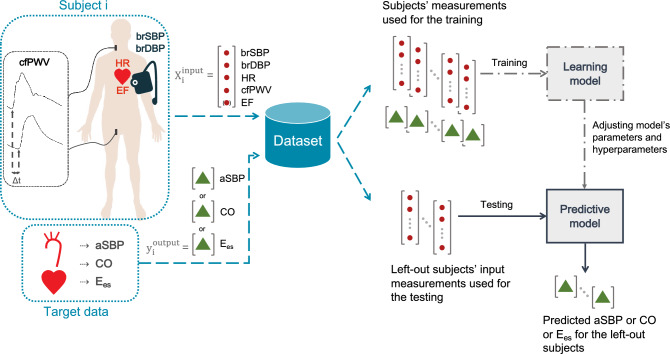
Schematic illustration of the regression pipeline. *brSBP* Brachial systolic blood pressure, *brDBP* brachial diastolic blood pressure, *HR* heart rate, *cfPWV* carotid-to-femoral pulse wave velocity, and *EF* ejection fraction were used as features for predicting aortic systolic blood pressure (aSBP), cardiac output (CO), and end-systolic elastance (E_es_). Regression models were trained to map the input data to the respective target data of interest. The methodology presented here was followed for each regression process (in terms of set of inputs, model, and output).

The present study aimed to evaluate whether aortic hemodynamics (i.e., aSBP and CO) and cardiac contractility (i.e., E_es_) can be accurately predicted by the use of brachial systolic blood pressure (brSBP) and diastolic blood pressure (brDBP), heart rate (HR), carotid-to-femoral pulse wave velocity (cfPWV), and, if necessary, ejection fraction (EF). These quantities were chosen as they are readily available in clinical practice and have been shown to provide information on the cardiovascular state^2–4,32^. To overcome the aforementioned limitations, we performed our experiments using synthetic data (n = 4,018), which were generated using a previously validated one-dimensional (1-D) mathematical model of the cardiovascular system^28^. Regression analysis was performed to establish the relationship between the noninvasive measurements (brSBP, brDBP, HR, cfPWV, (and EF)) and the invasive quantities of interest (aSBP, CO, and E_es_). The regression pipeline of the present study is presented in Fig. 1. A ten-fold cross validation (CV) scheme was employed for the training/testing of the proposed approach. We evaluated four models including Random Forest^33^, Support Vector Regressor (SVR)^34^, Ridge^35^, and Gradient Boosting^36^. In addition, averaging of the multiple predictions was performed. Two approaches were investigated: (i) prediction of aSBP, CO, and E_es_ using brSBP, brDBP, HR, and cfPWV as inputs, and (ii) prediction of E_es_ using brSBP, brDBP, HR, cfPWV, and EF. The accuracy of our prediction was evaluated by comparing the model-derived values with the reference simulated data. The accuracy of the aSBP model was subsequently validated using a large clinical dataset including in-vivo hemodynamic measurements (n = 783). Lack of CO and E_es_ in-vivo data impeded the clinical evaluation of the corresponding models.

## Results

Table 1 aggregates the cardiovascular parameters of the in-silico study population. The comparisons between the model-derived predictions and the reference data are presented below for each of the targeted outputs.

**Table 1:** Distributions of the parameters of the in-silico population (n = 4,018).

Parameter	Value (n = 4,018)			
	Min	Max	Mean	SD
End-systolic elastance (mmHg mL^−1^)	1.03	3.50	2.29	0.40
End-diastolic elastance (mmHg mL^−1^)	0.05	0.20	0.12	0.09
Filling pressure (mmHg)	7.00	23.00	15.12	2.10
Total arterial compliance (mL mmHg^−1^)	0.10	3.80	1.86	0.90
Total peripheral resistance (mmHg s mL^−1^)	0.50	1.30	0.80	0.19
Heart rate (bpm)	61.11	10.00	82.57	8.15
Aortic diameter (cm)	2.00	4.00	3.00	1.00
Height (cm)	150.00	200.00	175.00	25.00
Brachial systolic blood pressure (mmHg)	81.80	199.20	133.71	25.07
Brachial diastolic blood pressure (mmHg)	39.73	125.69	76.06	21.86
Aortic systolic blood pressure (mmHg)	76.05	188.31	121.71	24.96
Carotid-to-femoral pulse wave velocity (m s^−1^)	5.53	14.27	8.89	1.63
Cardiac output (L min^−1^)	3.26	10.56	5.94	1.22
Ejection fraction (%)	29.74	69.31	50.83	6.81

### Prediction of aSBP, CO, and E_es_ from brSBP, brDBP, HR, and cfPWV

For the four models, the comparison between the predicted aSBP and the actual aSBP is presented in Table 2. The average difference (in absolute value) between the model-aSBP and the reference aSBP was less than 5 mmHg in 87% of the total cases for Random Forest, 89% for SVR, 75% for Ridge, and 88% for Gradient Boosting, respectively. Accuracy, correlation and agreement of model-CO estimates in comparison to the reference data are summarized in Table 3. The difference between model-CO and reference CO was less than 0.3/0.5 L min^−1^ in 62/84% of the population for Random Forest, 65/86% for SVR, 50/74% for Ridge, and 63/85% for Gradient Boosting. Finally, the E_es_ predictions are compared to the reference data in Table 4. High errors were reported for all of the regression models, whereas correlation between the estimated and the reference data was significantly poor.

**Table 2:** Regression statistics between model predicted aSBP and reference aSBP.

Model	Slope	Intercept (mmHg)	r	R^2^	p-value	RMSE (mmHg)	nRMSE (%)	MAE (mmHg)
Random forest	1.01	− 1.13	0.99	0.98	< 0.001	3.33 ± 1.16	3.57 ± 0.79	2.61 ± 0.87
SVR	1.01	− 1.00	0.99	0.98	< 0.001	3.13 ± 1.06	3.36 ± 0.74	2.43 ± 0.77
Ridge	0.99	1.64	0.98	0.96	< 0.001	4.55 ± 2.17	4.96 ± 2.04	3.73 ± 1.71
Gradient boosting	1.01	− 0.87	0.99	0.98	< 0.001	3.31 ± 1.22	3.55 ± 0.88	2.58 ± 0.90
Ensemble averaging (all)	1.01	− 0.85	0.99	0.98	< 0.001	3.31 ± 1.35	3.53 ± 1.00	2.59 ± 1.01
Ensemble averaging (RF, SVR, GB)	1.01	− 1.13	0.99	0.98	< 0.001	3.17 ± 1.13	3.40 ± 0.79	2.47 ± 0.84

The input features include brSBP, brDBP, HR, and cfPWV.

*r* correlation coefficient; *R*^*2*^ coefficient of determination; *RMSE* root mean squared error; *nRMSE* normalized RMSE; *MAE* mean absolute error; *RF* random forest; *SVR* support vector regressor; *GB* gradient boosting.

**Table 3:** Regression statistics between model predicted CO and reference CO.

Model	Slope	Intercept (L min^−1^)	r	R^2^	p-value	RMSE (L min^−1^)	nRMSE (%)	MAE (L min^−1^)
Random forest	0.99	0.03	0.95	0.90	< 0.001	0.36 ± 0.10	7.94 ± 0.95	0.29 ± 0.08
SVR	1.01	− 0.06	0.96	0.92	< 0.001	0.34 ± 0.08	7.60 ± 0.68	0.27 ± 0.06
Ridge	0.99	0.05	0.93	0.86	< 0.001	0.45 ± 0.07	10.15 ± 1.00	0.36 ± 0.05
Gradient boosting	1.00	0.01	0.95	0.90	< 0.001	0.35 ± 0.09	7.80 ± 0.86	0.28 ± 0.07
Ensemble averaging (all)	1.02	− 0.11	0.96	0.92	< 0.001	0.34 ± 0.08	7.59 ± 0.72	0.27 ± 0.06
Ensemble averaging (RF, SVR, GB)	1.01	− 0.05	0.96	0.92	< 0.001	0.34 ± 0.08	7.48 ± 0.73	0.27 ± 0.06

The input features include brSBP, brDBP, HR, and cfPWV.

*r* correlation coefficient; *R*^*2*^ coefficient of determination; *RMSE* root mean squared error; *nRMSE* normalized RMSE; *MAE* mean absolute error; *RF* random forest; *SVR* support vector regressor; *GB* gradient boosting.

**Table 4:** Regression statistics between model predicted E_es_ and reference E_es_.

Model	Slope	Intercept (mmHg mL^−1^)	r	R^2^	p-value	RMSE (mmHg mL^−1^)	nRMSE (%)	MAE (mmHg mL^−1^)
Random forest	0.93	0.17	0.36	0.13	< 0.001	0.38 ± 0.02	17.02 ± 0.63	0.30 ± 0.02
SVR	0.87	0.30	0.35	0.12	< 0.001	0.38 ± 0.02	17.11 ± 0.67	0.30 ± 0.02
Ridge	1.00	− 0.00	0.37	0.14	< 0.001	0.37 ± 0.02	16.96 ± 0.64	0.30 ± 0.02
Gradient boosting	0.99	0.02	0.33	0.10	< 0.001	0.38 ± 0.02	17.23 ± 0.72	0.31 ± 0.02
Ensemble averaging (all)	1.01	− 0.02	0.37	0.14	< 0.001	0.38 ± 0.02	16.98 ± 0.65	0.30 ± 0.02
Ensemble averaging (RF, SVR, GB)	0.99	0.02	0.36	0.13	< 0.001	0.38 ± 0.02	17.02 ± 0.66	0.30 ± 0.02

The input features include brSBP, brDBP, HR, and cfPWV.

*r* correlation coefficient; *R*^*2*^ coefficient of determination; *RMSE* root mean squared error; *nRMSE* normalized RMSE; *MAE* mean absolute error; *RF* random forest; *SVR* support vector regressor; *GB* gradient boosting.

### Prediction of E_es_ from brSBP, brDBP, HR, cfPWV, and EF

The statistics of the second regression analysis for E_es_, i.e., after additional knowledge of EF, are presented in Table 5. Differences between the predicted E_es_ and the actual E_es_ were found to be less than 0.05/0.20 mmHg mL^−1^ in the 47/78%, 51/81%, 39/70%, and 47/78% of the entire population, for Random Forest, SVR, Ridge, and Gradient Boosting, respectively.

**Table 5:** Regression statistics between model predicted E_es_ and reference E_es_.

Model	Slope	Intercept (mmHg mL^−1^)	r	R^2^	p-value	RMSE (mmHg mL^−1^)	nRMSE (%)	MAE (mmHg mL^−1^)
Random forest	1.02	− 0.04	0.91	0.83	< 0.001	0.17 ± 0.02	7.57 ± 0.92	0.13 ± 0.02
SVR	1.00	0.00	0.92	0.85	< 0.001	0.15 ± 0.02	7.00 ± 0.78	0.12 ± 0.01
Ridge	0.97	0.06	0.87	0.76	< 0.001	0.20 ± 0.03	9.04 ± 1.36	0.16 ± 0.03
Gradient boosting	1.00	− 0.01	0.91	0.83	< 0.001	0.16 ± 0.02	7.43 ± 0.81	0.13 ± 0.01
Ensemble aaveraging (all)	1.03	− 0.08	0.92	0.85	< 0.001	0.16 ± 0.02	7.20 ± 0.76	0.13 ± 0.01
Ensemble averaging (RF, SVR, GB)	1.02	− 0.05	0.92	0.85	< 0.001	0.16 ± 0.01	7.04 ± 0.70	0.12 ± 0.01

The input features include brSBP, brDBP, HR, cfPWV, and EF.

*r* correlation coefficient; *R*^*2*^ coefficient of determination; *RMSE* root mean squared error; *nRMSE* normalized RMSE; *MAE* mean absolute error; *RF* random forest; *SVR* support vector regressor; *GB* gradient boosting.

**Figure 2:**
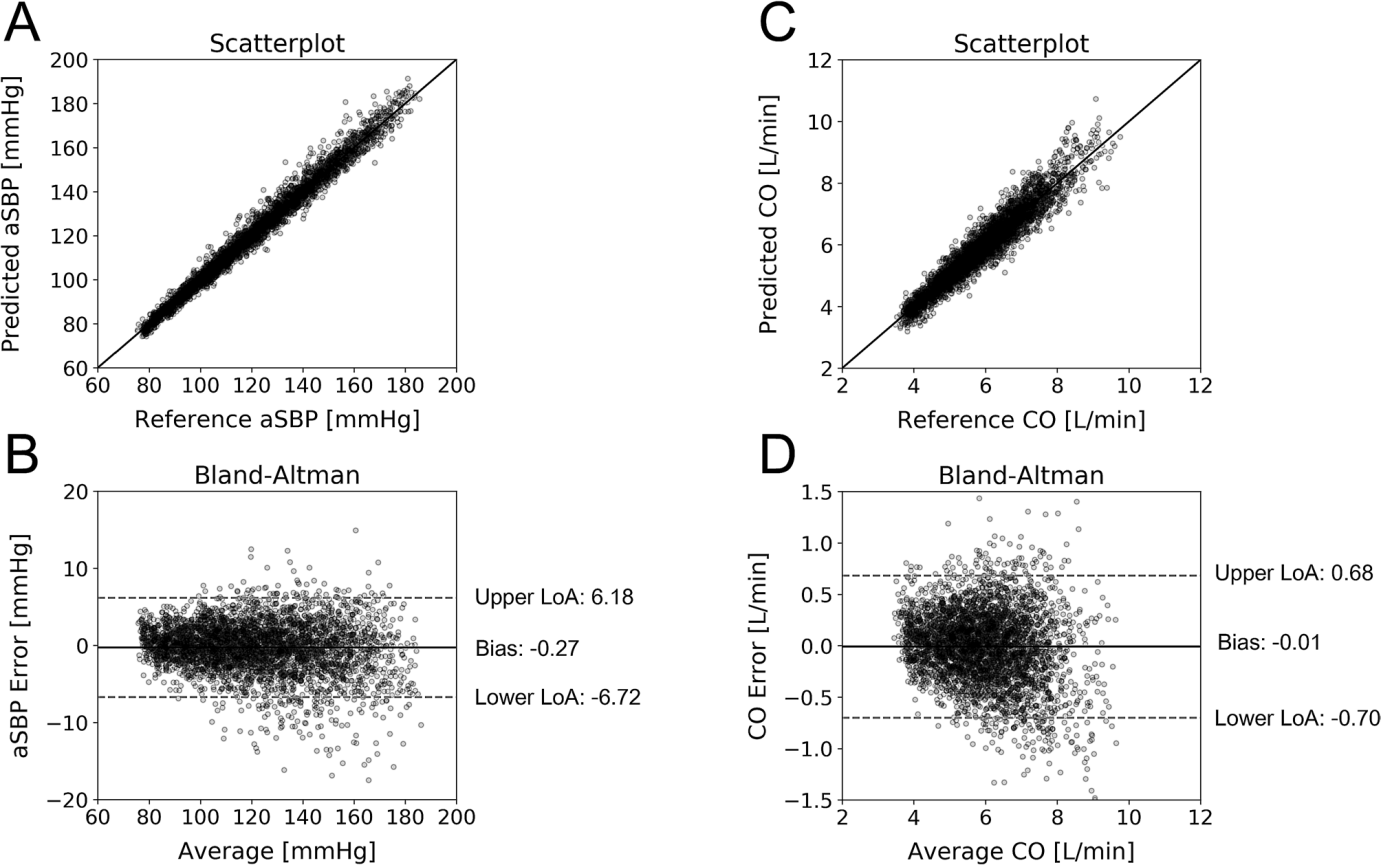
Comparison between predicted and reference data. Scatterplots and Bland–Altman plots between: (**A**, **B**) the predicted aSBP and the reference aSBP, and (**C**, **D**) the predicted CO and the reference CO. The solid line of the scatterplots represents equality. In Bland–Altman plots, limits of agreement (LoA), within which 95% of errors are expected to lie, are defined by the two horizontal dashed lines.

**Figure 3:**
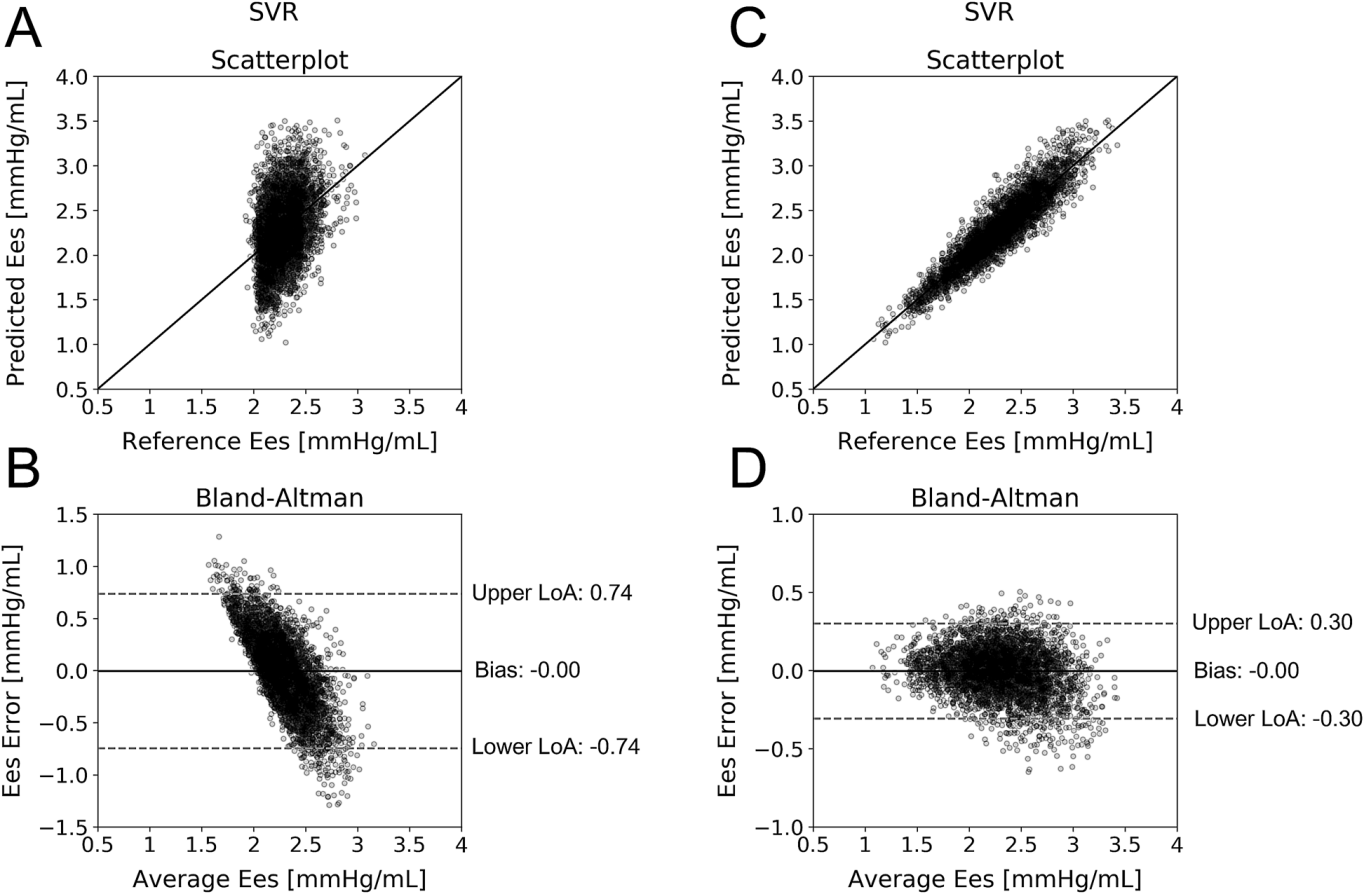
Comparison between predicted and reference data. Scatterplots and Bland–Altman plots between: (**A**, **B**) the predicted E_es_ and the reference E_es_ without ejection fraction as regression input, and (**C**, **D**) the predicted E_es_ and the reference E_es_ with ejection fraction as regression input. The solid line of the scatterplots represents equality. In Bland–Altman plots, limits of agreement (LoA), within which 95% of errors are expected to lie, are defined by the two horizontal dashed lines.

The scatterplots and Bland–Altman graphs for the best performing models are provided in Figs. 2 and 3. The plotted data are corrupted with random noise (see “Blending the dataset with random noise” in “Methods”). Table 6 presents the frequency of selection for each hyperparameter value over the tenfold CV for the best performing model. For the aSBP and E_es_ estimators, we observed an apparent consistency for the values of the *C* and *gamma* hyperparameters. Concretely, *C* and *gamma* were set at 100 and 0.001 for aSBP, and 10 and 0.001 for E_es_, respectively, in the totality of the 10 folds. Such a consistency is not evident for the CO estimator where *C* was set at 100 for the 60% of the times. Nevertheless, *gamma* was again consistently selected to be 0.001.

**Table 6:** Statistical results in percentage of times that the hyperparameter value was selected during the hyperparameter tuning with tenfold cross validation process.

Model	Hyperparameters	Values	aSBP	CO	E_es_
			Times selected (%)	Times selected (%)	Times selected (%)
**SVR**	*C*	1	0	0	0
		10	0	40	**100**
		100	**100**	60	0
	*gamma*	0.001	**100**	**100**	**100**
		0.01	0	0	0
		0.1	0	0	0
		1	0	0	0

Values selected consistently are presented in bold.

*aSBP* aortic systolic blood pressure; *CO* cardiac output; *E*_*es*_ end-systolic elastance; *SVR* support vector regressor.

### Sensitivity analysis for the training size

**Figure 4:**
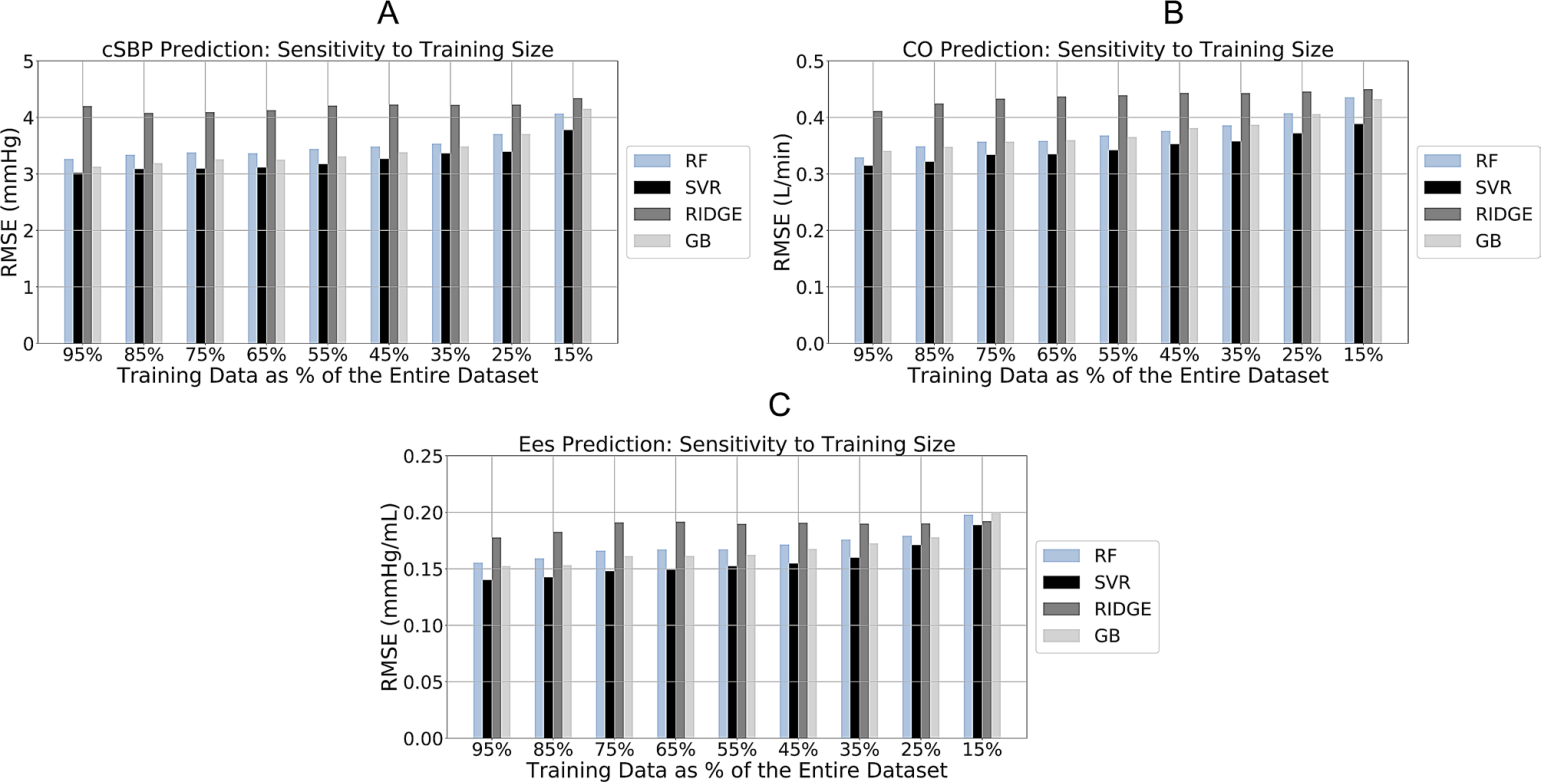
Sensitivity of RMSE to changes on the training size for aortic systolic blood pressure (aSBP) (**A**), cardiac output (CO) (**B**), and end-systolic elastance (E_es_) **(C).** *RMSE* root mean square error; *RF* random forest; *SVR* support vector regressor; *GB* gradient boosting.

The training size, that is, the number of data instances used for training, plays a major role on the accuracy of the predictions. To investigate the sensitivity to the number of training data, the training size was modified from 95 to 15% of the total number of cases (Fig. 4). For all models except for Ridge, the RMSEs were increased gradually with decreasing training size. For the Random Forest, SVR, and Gradient Boosting, the RMSEs of the aSBP predictions were less than 4.20 mmHg. Using Ridge, the RMSE varied at a lesser extent, while it was consistently higher compared to the rest of the models. For the CO predictions, all RMSE values were less than 0.50 L min^−1^. In particular, RMSE for SVR did not exceed 0.38 L min^−1^, even when only the 15% of the entire population was used for the training. Finally, all RMSEs of E_es_ estimations were equal or below 0.20 mmHg mL^−1^.

### Feature importance evaluation

**Figure 5:**
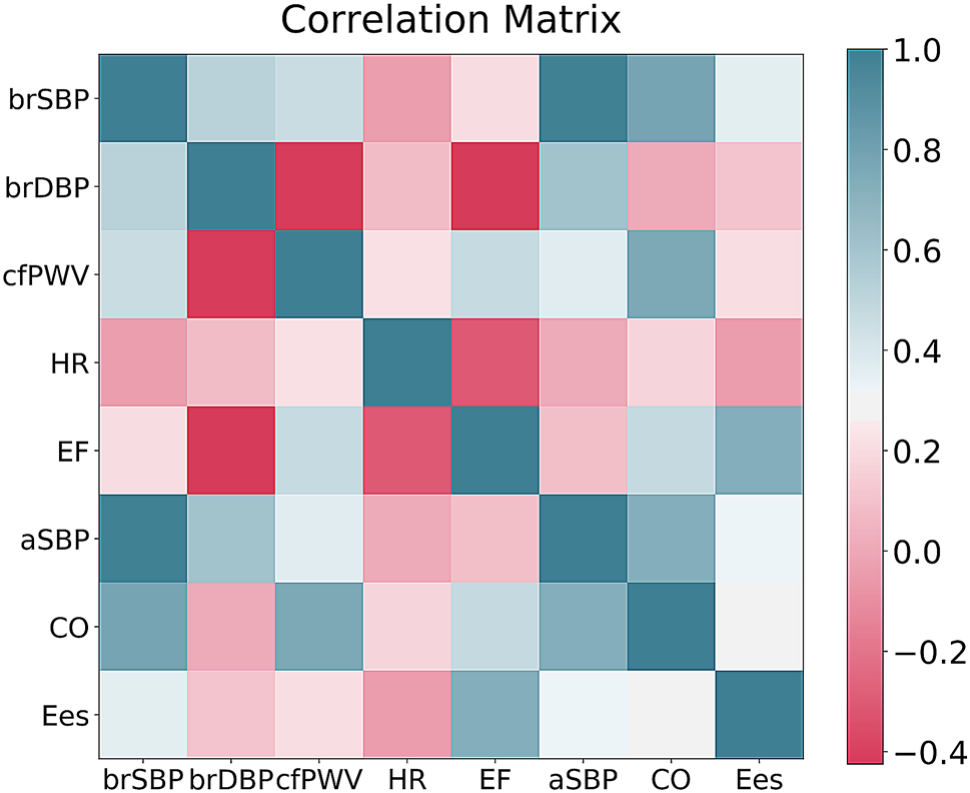
Correlation matrix for the in-silico database.

Figure 5 presents the correlation matrix reporting the inter-feature correlations, and the correlations between the inputs and the target outputs. Table 7 presents the average importances of the input features, sorted in a descending order for predicting aSBP, CO, and E_es_, respectively. For estimating aSBP, brSBP was found to be a critical contributor; the importance level (0.98) indicated that brSBP should be sufficient for estimating aSBP. The features of brSBP and cfPWV were the dominant contributors in the estimation of CO. Finally, EF was found to play the most significant role in the E_es_ prediction, followed by brDBP and HR. To further verify the sensitivity of the model’s performance to the input features, we present the RMSE variation for different subsets of input features (only for the best performing models) (Table 8).

**Table 7:** Average feature importances for the prediction of aSBP, CO, and E_es_.

Feature	aSBP	Feature	CO	Feature	E_es_
brSBP	0.98	brSBP	0.54	EF	0.65
brDBP	0.02	cfPWV	0.33	brDBP	0.16
HR	0.004	brDBP	0.08	HR	0.11
cfPWV	0.003	HR	0.04	cfPWV	0.05
				brSBP	0.02

*aSBP* aortic systolic blood pressure; *CO* cardiac output; *E*_*es*_ end-systolic elastance; *brSBP* brachial systolic blood pressure; *brDBP* brachial diastolic blood pressure; *HR* heart rate; *cfPWV* carotid-to-femoral pulse wave velocity; *EF* ejection fraction.

**Table 8:** Model performance for the best performing configurations (SVR) using different subsets of the input features.

Input features’ subsets	RMSE (r)		
	aSBP (SVR)	CO (SVR)	Ees (SVR)
brSBP, brDBP, HR, cfPWV, EF	–	–	0.15 mmHg mL^−1^ (0.92)
brSBP, brDBP, HR, EF	–	–	0.17 mmHg mL^−1^ (0.91)
brSBP, brDBP, cfPWV, EF	–	–	0.17 mmHg mL^−1^ (0.91)
brSBP, HR, cfPWV, EF	–	–	0.22 mmHg mL^−1^ (0.83)
brDBP, HR, cfPWV, EF	–	–	0.17 mmHg mL^−1^ (0.91)
brSBP, brDBP, HR, cfPWV	3.13 mmHg (0.99)	0.34 L min^−1^ (0.96)	0.37 mmHg mL^−1^ (0.37)
brSBP, brDBP, HR	3.31 mmHg (0.99)	0.38 L min^−1^ (0.95)	0.38 mmHg mL^−1^ (0.33)
brSBP, brDBP, cfPWV	3.09 mmHg (0.99)	0.42 L min^−1^ (0.93)	0.38 mmHg ^−1^ (0.35)
brSBP, HR, cfPWV	3.88 mmHg (0.99)	0.59 L min^−1^ (0.85)	0.38 mmHg mL^−1^ (0.35)
brDBP, HR, cfPWV	7.68 mmHg (0.94)	0.59 L min^−1^ (0.86)	0.38 mmHg mL^−1^ (0.32)

*RMSE* root mean squared error; *r* correlation coefficient; *SVR* support vector regressor; *aSBP* aortic systolic blood pressure; *CO* cardiac output; *E*_*es*_ end-systolic elastance; *brSBP* brachial systolic blood pressure; *brDBP* brachial diastolic blood pressure; *HR* heart rate; *cfPWV* carotid-to-femoral pulse wave velocity.

For aSBP, it was shown again that the brSBP is the most pivotal predictor of aSBP; when brSBP was removed from the input features, the RMSE increased significantly. On the contrary, a precise prediction of CO requires the use of at least one of the brachial BP values; exclusion of the latter resulted to a deterioration of the model’s performance. Finally, E_es_ appears to be mainly sensitive to EF which significantly contributes to the accuracy of the E_es_ estimation. Results of the hypothesis testing for the ordinary least squares (OLS) regression coefficients are summarized in Table 9. All of the specified coefficients were statistically significantly different from zero.

**Table 9:** The *t*-statistics for the OLS regression coefficients.

Input feature	aSBP		CO		E_es_	
	t-values	p-values	t-values	p-values	t-values	p-values
Intercept	− 31.296	< 0.001	− 22.304	< 0.001	− 60.951	< 0.001
brSBP	148.210	< 0.001	82.000	< 0.001	− 12.704	< 0.001
brDBP	11.241	< 0.001	− 51.739	< 0.001	32.673	< 0.001
cfPWV	− 9.087	< 0.001	− 18.746	< 0.001	3.685	< 0.001
HR	16.776	< 0.001	47.129	< 0.001	21.960	< 0.001
EF	–	–	–	–	118.028	< 0.001

*aSBP* aortic systolic blood pressure; *CO* cardiac output; *E*_*es*_ end-systolic elastance; *brSBP* brachial systolic blood pressure; *brDBP* brachial diastolic blood pressure; *HR* heart rate; *cfPWV* carotid-to-femoral pulse wave velocity; *EF* ejection fraction.

### In-vivo evaluation of the aSBP estimations

After the in-silico validation, the performance of the aSBP estimator was evaluated anew using clinical data. The population included both women (n = 136) and men (n = 647). The descriptive and clinical characteristics of the clinical population are presented in Table 10.

**Table 10:** Distributions of the parameters of the in-vivo population (n = 783).

Parameter	Value (n = 783)			
	Min	Max	Mean	SD
Age (years)	28.00	88.00	60.83	11.47
Height (kg)	143.00	195.00	171.60	7.94
Weight (kg)	40.00	145.00	82.29	14.10
Heart rate (bpm)	41.00	107.00	64.06	10.65
Brachial systolic blood pressure (mmHg)	90.00	180.00	126.37	15.70
Brachial diastolic blood pressure (mmHg)	40.00	120.00	77.89	11.21
Central systolic blood pressure (mmHg)	82.00	172.00	117.95	15.18
Carotid-to-femoral pulse wave velocity (m s^−1^)	4.70	19.60	8.92	2.25
Hypertension	64%			
Dyslipidemia	64%			
Smoking^a^	23%			
Renal transplant LD	1%			
Renal transplant DD	0.3%			
Breast cancer	2%			
Coronary artery disease	81%			

^a^65% of the remaining population declared to be smokers in the past.

**Figure 6:**
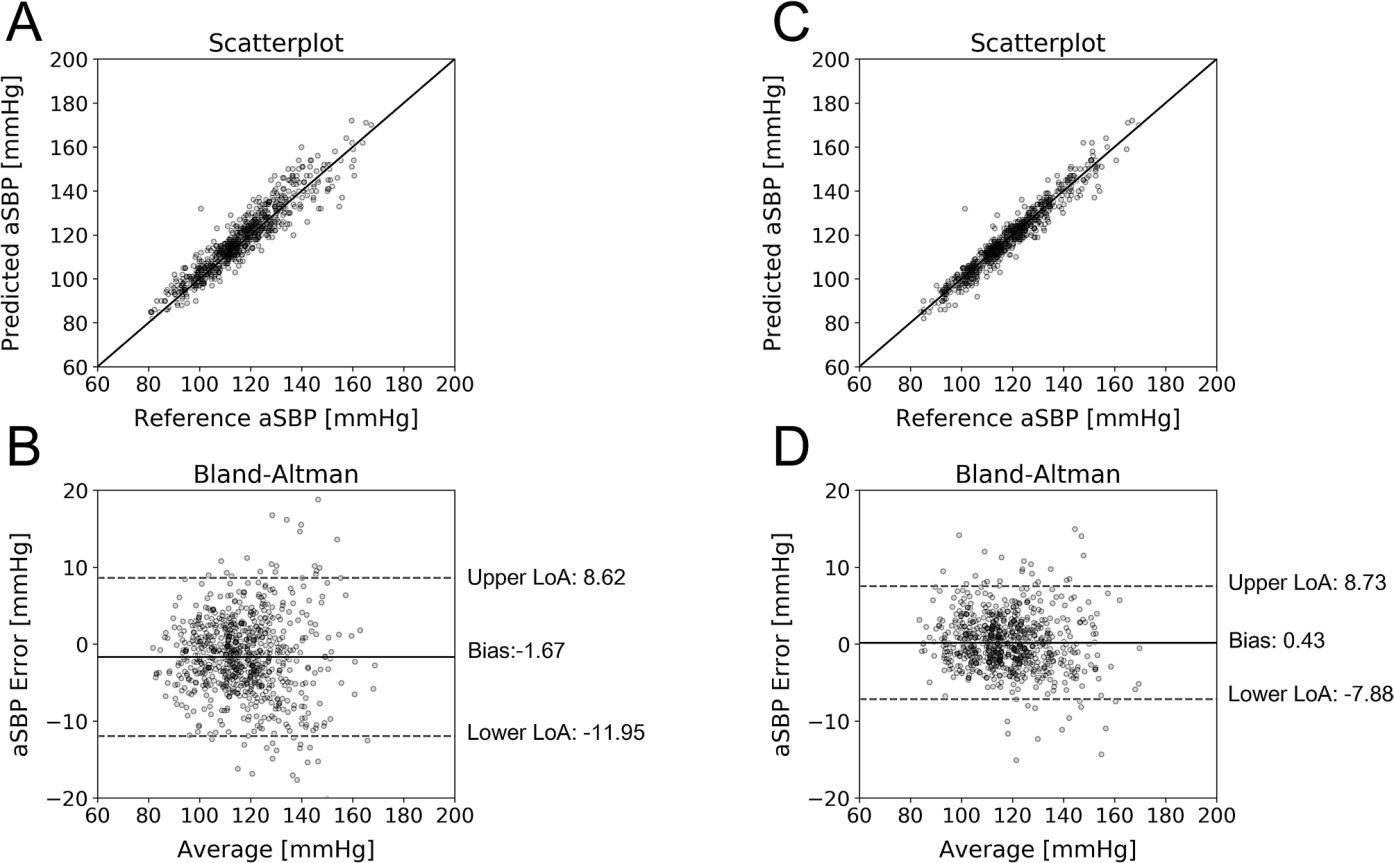
Comparison between predicted and reference clinical data. Scatterplots and Bland–Altman plots between: the predicted aSBP and the reference aSBP for SVR trained using in-silico data (**A**, **B**), and for SVR trained using in-vivo data (**C**, **D**). The solid line of the scatterplots represents equality. In Bland–Altman plots, the limits of agreement (LoA), within which 95% of errors are expected to lie, are defined by the two horizontal dashed lines.

**Figure 7:**
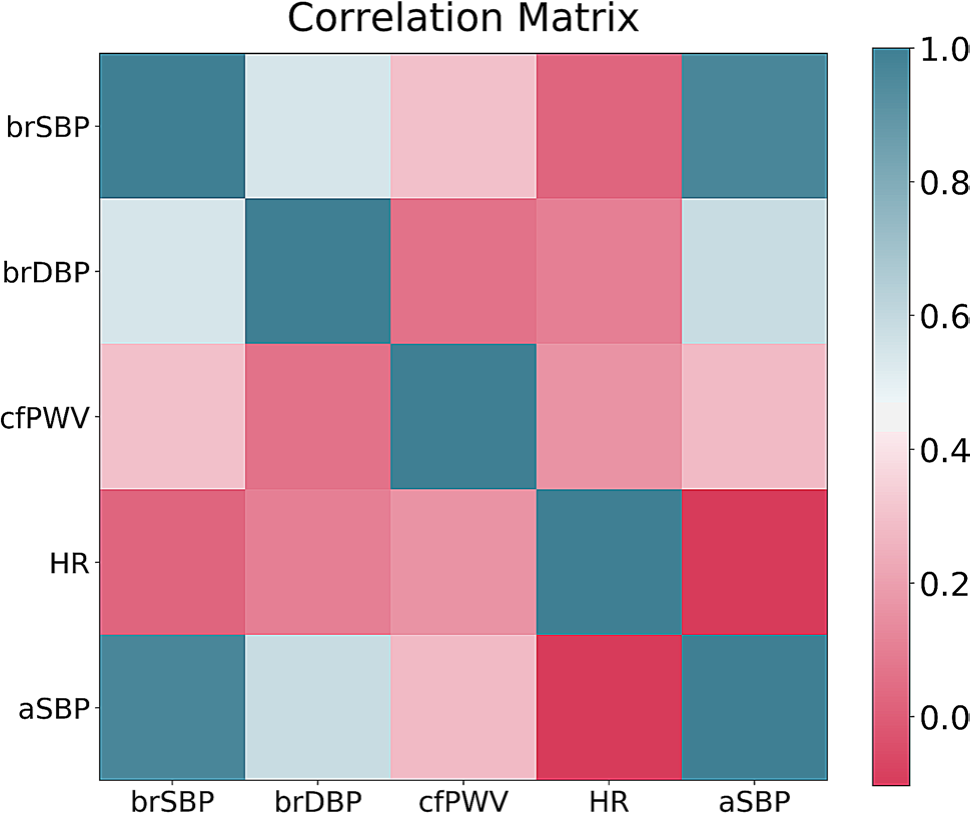
Correlation matrix for the in-vivo database.

The comparisons between the predicted aSBP and the reference aSBP are presented below. First, we assessed the capacity of an SVR model, which was trained using only in-silico data, to make an accurate prediction for the human population (Fig. 6A,B). Then, we compared the latter’s performance with an SVR model which was trained using in-vivo data (Fig. 6C,D). The regression statistics between the model predictions and the reference data are summarized in Table 11. For the in-vivo data, the hypothesis testing’s results for the OLS regression coefficients are presented in Table 12. Figure 7 provides the correlation matrix for the in-vivo dataset.

**Table 11:** Regression statistics between model predicted aSBP and reference aSBP.

SVR (tested using in-vivo data)	Slope	Intercept (mmHg)	r	R^2^	p-value	RMSE (mmHg)	nRMSE (%)	MAE (mmHg)
Model trained using in-silico data	0.99	2.94	0.94	0.88	< 0.001	5.34	5.93	4.10
Model trained using in-vivo data	1.00	0.31	0.97	0.94	< 0.001	3.53 ± 1.27	5.26 ± 2.30	2.74 ± 1.14

The input features include brSBP, brDBP, HR, and cfPWV.

The testing set consists of in-vivo data only.

*R*^*2*^ coefficient of determination; *r* correlation coefficient; *RMSE* root mean squared error; *nRMSE* normalized RMSE; *MAE* mean absolute error; *SVR* Support Vector Regressor; *n.s.* not significant; *SD* standard deviation.

**Table 12:** The *t*-statistics for the OLS regression coefficients.

Input feature	aSBP	
	t-values	p-values
Intercept	6.504	< 0.001
brSBP	91.182	< 0.001
brDBP	12.094	< 0.001
cfPWV	3.296	< 0.001
HR	− 18.110	< 0.001

*aSBP* aortic systolic blood pressure; *brSBP* brachial systolic blood pressure; *brDBP* brachial diastolic blood pressure; *HR* heart rate; *cfPWV* carotid-to-femoral pulse wave velocity

## Discussion

The present study demonstrated that accurate estimations of central hemodynamics (namely, aSBP and CO) and left ventricular E_es_ from readily available noninvasive clinical measurements can be obtained by using machine learning models. Our basic hypothesis was whether brSBP, brDBP (cuff BP), HR, and cfPWV provide sufficient information to predict aSBP, CO, and E_es_. However, for the determination of E_es_, data from peripheral pressure waves fall short to provide a precise estimate. Our results indicated that additional information, such as the EF, which is directly measured in the heart (rather than the periphery) may improve the noninvasive E_es_ predictions. To our best knowledge, this is the first work to evaluate the use of machine learning models in predicting cardiac contractility.

The best performing prediction model for all three target outputs was SVR which outperformed the other models accomplishing the highest accuracy. The E_es_ estimation was effectively achieved only with the inclusion of EF in the set of input features. In order to evaluate the robustness of our regression models, sensitivity to the training size was investigated. The RMSE was gradually increased with decreasing the number or training samples for Random Forest, SVR, and Gradient boosting. Variations were less distinct for Ridge. Despite the increase in RMSE with changes in the training size, the errors lied within acceptable limits^37–41^ for Random Forest, SVR, and Gradient Boosting.

Moreover, we tested the performance of an ensemble predictor which used averaging of the single models’ predictions. The ensemble prediction model did not outperform the best performing single prediction model (SVR). However, such an approach may benefit the estimations’ accuracy by reducing the variance of the predictor and thus may improve the model’s generalization ability^42^. To avoid overwhelm the reader with an exhaustive report of several other approaches, we did not explore other ensemble learning techniques. Such an extensive exploration of different ensemble techniques would be out of the scope of this study.

Following the in-silico validation, in-vivo validation was performed only for the aSBP. The aSBP predictions were found to be precise in the both investigated scenarios, i.e., SVR trained with in-silico data, and SVR trained with in-vivo data. The accuracy was slightly higher in the second scenario despite the smaller size of the training dataset. This is expected if we consider that the in-vivo data may contain more physiologically relevant content and thus be more informative compared to the in-silico data in the training of the model. Interestingly, the hyperparameter tuning led to the same selection for the hyperparameters *C* = 100 (selected 100% of the times) and *gamma* = 0.001 (selected 100% of the times) when the SVR model was validated using the in-vivo population. These findings may verify that the in-silico predictive models can be rather informative for the design of clinical studies.

The principal reason that brSBP, brDBP, HR, and cfPWV were selected as the model inputs was the simplicity of their measurement in a clinical setting. Brachial cuff pressure constitutes a readily available and cost-efficient measurement in traditional medicine. At the same time, the use of pressure-based cfPWV is steadily increasing, as a result of numerous studies demonstrating its importance as an independent predictor of cardiovascular disease^43–45^. The convenience and the cost-efficiency of the aforementioned measurements render them suitable for easy, noninvasive, regular medical check-ups.

Based on the feature importances’ assessment, the aSBP prediction was found to be determined mainly from the brSBP. The strong dependency between aSBP and brSBP errors is to be expected, given that the two values are strongly related to mean BP, which is practically the same in both the aorta and the brachial artery. Moreover, brSBP seemed to be a significant predictor of CO. Resistance, and thus mean BP, is a strong determinant of CO. Given that brSBP is related to mean BP, this means that brSBP is indirectly related to CO. In addition, cfPWV is a measure of arterial compliance, which is also determinant of stroke volume and thus CO. Finally, EF and E_es_ have been reported to be positively correlated^46^ and this further explains the high importance level of EF for predicting E_es_. The results using different subsets of the input features further verified each feature’s contribution to the predictions of the target output variables.

Prior work proposed by Xiao et al.^47^ used an artificial neural network (ANN) to predict aSBP from invasive radial SBP and DBP, and HR. The differences between the predicted aSBP and the measured aSBP were found to be low and equal to − 0.30 ± 5.90 mmHg. Despite providing accurate results, invasive radial BP is not commonly measured on a regular basis, and thus its modelling imposes a substantial limitation on the clinical application of their proposed model. When an ANN with the same configuration, as the one reported in the study of Xiao et al., was employed to estimate aSBP in our study, the results indicated a similarly good prediction performance. Concretely, the employment of the ANN using only the in-silico data (n = 4,018) achieved an RMSE = 3.79 ± 1.88 mmHg and r = 0.99 (p < 0.001). Training/testing the ANN with only the in-vivo data (n = 783) achieved an RMSE = 3.38 ± 1.09 and r = 0.97 (p < 0.001). In the case of the in-vivo data, we observed that the accuracy is slightly improved by the use of ANN compared to the best performing configuration (SVR achieved an RMSE = 3.53 ± 1.27 mmHg, r = 0.97, p < 0.001).

In general, the majority of previous aSBP estimators relies on features extraction from the pressure waveforms^47,48^. In our approach, apart from peripheral SBP and DBP, and HR, we incorporated the cfPWV measurement. The idea was that cfPWV being an index of aortic stiffening would improve the performance of the model and strengthen the clinical relevance of our results. However, feature importances indicated that brSBP may be sufficient for estimating aSBP. Using only brSBP, brDBP, and HR as inputs would not alter significantly the accuracy of the estimation of aSBP (using the in-silico data); namely, the RMSE would slightly increase from 3.13 to 3.31 mmHg for the best performing model. In the case that only brSBP and brDBP were used as input features, the accuracy would deteriorate with a RMSE of 3.46 mmHg which could still be acceptable. The use of only brSBP as an input, however, would essentially increase the error at 5.33 mmHg. For the clinical dataset, the same errors were equal to 3.52 mmHg (brSBP, brDBP, HR as inputs) and 4.11 mmHg (brSBP, brDBP as inputs). Finally, using only the brSBP predictor would lead to an RMSE = 4.20 mmHg.

In addition to prediction models for aSBP, estimation of CO from arterial BP characteristics has been a fertile area of research. Dabanloo et al.^25^ has evaluated the performance of neural networks in predicting CO from invasive arterial pressure waves. Upon comparison between the predicted CO and thermodilution-derived CO, their best performing model provided a mean absolute error equal to 0.54 L min^−1^ and a correlation coefficient of 0.89. Nevertheless, their model made use of the entire pressure waveform, from which input features were extracted, whereas it provided only an uncalibrated estimation of CO rather than its absolute value.

The results presented in this study are also compliant with the findings of Bikia et al.^49^, who suggested that brachial BP and cfPWV can be used to predict central SBP and CO (RMSE equal to 2.46 mmHg and 0.36 L min^−1^, respectively). Following an inverse problem-solving approach, a generalized model of the cardiovascular system was adjusted to quasi- patient-specific standards using measurements of brSBP, brDBP, HR, and cfPWV. Additional geometrical information on the aortic diameter size of each subject was also integrated. The aortic diameter was approximated using previously published age- and BSA-related data^50^. A similar approximation of the aortic geometry could be embedded in the present study and improve the accuracy of the results. Therefore, employment of machine learning on clinical data could be further reinforced with the inclusion of additional input features such as age, height, and weight. However, given that the errors are already rather low, it is not anticipated that such an improvement would be of particular clinical significance.

Additionally, this study aimed to effectively predict E_es_ while utilizing a small number of required inputs. Chen et al.^26^ proposed a method to estimate E_es_ from cuff pressure, stroke volume, and EF. Their method provided accurate predictions of E_es_ with differences equal to 0.43 ± 0.50 mmHg mL^−1^. In contrast to Chen’s approach, we excluded stroke volume from our input vector and, on the other hand, we introduced cfPWV which constitutes an index of aortic stiffness and thus a powerful index of arterio-ventricular coupling^51^. In an attempt to remove EF from the set of inputs, E_es_ was found to be poorly predicted. This underachieving performance may be rather expected given that a specific combination of brachial SBP and DBP, and cfPWV might not be unique for only a particular E_es_ value. Importantly, our study emphasized on the significance of EF in accurately predicting E_es_.

The use of EF is further encouraged from the fact that EF constitutes a noninvasive parameter which can be derived via several cardiac imaging modalities. The Simpson’s method^52^ has been the most commonly used technique; however, it might underestimate EF when compared to the magnetic resonance imaging (MRI), which has been shown to be the gold standard noninvasive technique for assessing LV function and thus EF^53^. Of course, the latter are not considered as convenient and cost-efficient as a cuff- or tonometry-based pressure measurement. It is likely that the EF-related information may be derived from another measured parameter which is directly or indirectly related to the cardiac contractility, e.g. electrical signals of cardiac events^54^. Further investigation towards this direction will be conducted in future work.

It should be noted that the aim of the current study is not to propose necessarily a tool that could provide simultaneous predictions of aSBP, CO, and E_es_. The models developed in this study could be considered as independent predictors for each of the target parameters in different clinical occasions. In particular, aSBP and CO are major hemodynamical indices that are often useful to the clinician and their noninvasive estimation is highly desirable in a routine clinical examination. On the other hand, E_es_ is less often required. Currently, E_es_ is measured invasively with the acquisition of the left ventricular pressure–volume loops. The invasive nature of this technique severely limits the use of E_es_ in clinical practice.

The booming of data has led to efforts of transferring one type of information to another using machine learning models. Specifically, in relation to patho-physiology, the advancement on measuring and imaging techniques has encouraged the employment of machine learning for estimating clinical pathophysiological indices and validating their results. This promising area of research could not exclude applications on cardiovascular health^25,47,55,56^. High correlation between peripheral pressure and central aortic pressure indicates the potential to estimate the latter from the former. However, the correlations for a complete set of cardiovascular variables have not been thoroughly investigated. In this work, we performed a first study to elucidate which input parameters (noninvasive measurements) are considered necessary when machine learning is employed for predicting aortic hemodynamics and contractility index (invasive measurements). A major advantage of the present study pertains to the well-balanced dataset that was used for the training/testing scheme. The use of synthetic data allowed for covering a wide range of hemodynamical characteristics, whereas it provided us with access to cardiovascular quantities which are difficult to obtain noninvasively in the real clinical setting, i.e., aortic BP or E_es_.

Cardiovascular models have attracted great interest due to the increasing impact of cardiovascular disease. They have provided a valuable alternative for the assessment of pressure and flow in the entire arterial network providing additional pathophysiological insights, which are difficult to acquire in-vivo. Numerous previous studies have used in-silico data for the estimation of aortic BP, cardiac output, aortic PWV and many more^56–60^. Importantly*,* in-silico studies allow for the preliminary evaluation of predictive models across a wide range of cardiovascular parameters^61^ in a quick and cost-efficient way, while their results can be rather informative of the design of clinical studies^62,63^.

Several limitations need to be acknowledged. The data used for the training/testing scheme were derived from a simulator instead of a real human population. While synthetic data can mimic numerous properties of the real clinical data, they do not copy the original content in an identical way. Nevertheless, the goal here was to define the minimum necessary input information that is required to estimate aortic hemodynamics and E_es_. Thus, despite that the use of synthetic data might not lead to exactly the same results with the results coming from clinical data, it should not undermine the reliability of the study’s findings. The latter has been verified by the in-vivo validation of our aSBP estimations. Clinical validation was not possible for the CO and E_es_ estimators, due to the lack of the respective data. At the initial stage of our research, we found it reasonable to start with an in-silico validation of our predictive models, instead of collecting measurements of CO and E_es_ in a large cohort. In addition, the cost and the complexity of the respective measurements would make it difficult to incorporate them in the current study. Future work should include the use of real-world data for all parameters that will finally verify the application of the proposed method in the clinical setting. Finally, the proposed models have been designed and tested on data coming from a generalized model of the cardiovascular system which was developed according to published data^28^. Hence, the precision of the predictions might be compromised in the case of pathological conditions, such as atherosclerosis, aneurysm or aortic valve disease. It is of great importance that in-vivo validation of the models should be conducted using pathological clinical data as well.

In summary, this study showed that the use of noninvasive arm-cuff pressure and PWV alone potentially allows for the estimation of aSBP and CO with acceptable accuracy. This might not be the case for E_es_ prediction. Nevertheless, the estimated E_es_ can be greatly improved when EF is used as an additional input in the prediction model. Following validation on in-vivo invasive data, this approach may provide a promising potential in the prediction of aortic hemodynamics and left ventricular contractility using unintrusive, readily available standard clinical measurements.

## Methods

A regression pipeline was applied for estimating aortic hemodynamics and LV contractility index. The schematic representation of the methodology is presented in Fig. 1. The input data comprised brSBP, brDBP, HR, cfPWV, and EF for every subject. These data were fed to the regression models to estimate aSBP, CO, and E_es_. First, brSBP, brDBP, HR, and cfPWV were used as input predictors for all three outputs, i.e., aSBP, CO, and E_es_. A second regression analysis was performed using EF as an additional input feature only for the estimation of E_es_. The outputs of each testing set were blinded and kept as the ground truth against which our predictions were later compared.

### Brief description of the in-silico model of cardiovascular dynamics

In the present study, we used a 1-D in-silico model of the cardiovascular system, that has been previously described and validated against in-vivo data^28,29^. The arterial tree includes the main arteries of the systemic circulation, as well as the cerebral circulation and the coronary circulation. In summary, the governing equations of the model are derived by integrating the longitudinal momentum and continuity equations over the arterial cross section. Pressure and flow are acquired across the arterial tree by solving the governing equations employing an implicit finite-difference scheme. Local arterial compliance is calculated, provided that pulse wave velocity (PWV) is approximated as an inverse power function of the arterial lumen diameter^28^. Three-element Windkessel models^64^ are coupled to the distal vessels to account for the peripheral resistance. The contractility of the LV is modeled using a time-varying elastance model^4,9^. This elastance model considers a linear ESPVR characterized by its slope, the end-systolic elastance (E_es_), and its intercept, the dead volume, V_d_, as well as a linear end-diastolic pressure–volume relation characterized by its slope, the end-diastolic elastance (E_ed_).

### Synthetic population generation

A database of 4,018 synthetic hemodynamic cases was created. The 1-D cardiovascular model ran using different combinations of arbitrary input parameters. The distributions of the input parameters were based on physiologically relevant data from the literature. The cardiovascular parameters were chosen to represent healthy individuals. Due to the limited amount of probabilistic information, the sampling was selected to be random Gaussian. The values of E_es_ and E_ed_ ranged within [1.03, 3.50] mmHg mL^−1^ and [0.05, 0.20] mmHg mL^−1^, respectively^65–67^. HR varied between 60 and 100 bpm. The LV filling pressure lied between 7.00 and 23.00 mmHg according to^68^. The dead volume (V_d_) and the time of maximal elastance (t_max_) were kept unchanged. Their selected values were equal to the mean values of V_d_ = 15.00 mL and t_max_ = 340.00 ms as reported by previously published works^28,69^. Arterial geometry was modified to simulate different body types by adapting the length and the diameter of the arterial vessels. The heights covered a range of [150.00, 200.00] cm while the limits for aortic diameter were set to [1.90, 4.00] cm^50,70^. Total peripheral resistance varied within 0.50–2.00 mmHg s mL^−1^^71^. Total arterial compliance was chosen within the range of [0.10, 3.80] mL mmHg^−1^ in order to account for a wide range of different values of arterial tree stiffness^72,73^. It should be noted that evidence of nonuniform aortic stiffening was integrated for the elderly and hypertensive virtual subjects, following the methodology described by Bikia et al.^49^.

### Virtual Database

The parameters of interest were estimated from the 1-D model-derived pressure and flow waves (simulation’s outputs). Concretely, synthetic brSBP, brDBP as well as HR data were obtained from the pressure wave at the left brachial artery. Similarly, aSBP was derived from the pressure waveform at the aortic root. CfPWV was derived using the tangential method^74^. The method computed the intersection (foot) of two tangents, i.e., the line passing tangentially through the systolic upstroke and the horizontal line passing through the point of minimum pressure. Subsequently, the pulse transit time was estimated between the foot of the wave at the two sites, namely, between the carotid artery and the femoral artery. The length between the two arterial sites was calculated by summing the lengths of the arterial segments within the transmission path. Finally, the cfPWV was estimated by dividing the arterial length of the path by the pulse transit time. Given that the ESPVR was known, the EF was derived by dividing the blood volume that is ejected within each heartbeat, i.e., the stroke volume (SV), by the end-diastolic volume (EDV). The value of the E_es_ was defined as the slope of the ESPVR. Then, all simulated information was discarded, except for the “measured” brSBP, brDBP, HR, cfPWV, and EF (inputs) and the aSBP, CO, and E_es_ data (outputs). The total dataset (organized in pairs of inputs and outputs) was kept for the training/testing process.

### Blending the dataset with random noise

The synthetic data were corrupted with random noise in order to represent a more realistic data collection. The introduced noise was equivalent to a random relative error within the range of [− 6.00, 6.00] % with respect to the actual value. This magnitude of error was selected based on published data from previous studies^75^.

### Clinical database

For the clinical validation of the aSBP estimations, we used clinical data from 783 subjects who underwent noninvasive cardiovascular assessment for research purposes, at the First University Department of Cardiology (Hippokration General Hospital, Athens, Greece). Anonymized data were analyzed in compliance with the Declaration of Helsinki of the World Medical Association and the National Regulations for clinical research.

The carotid-to-femoral pulse wave velocity (cfPWV) was measured in every subject as previously described^76–78^. In brief, cfPWV measurement was performed using the SphygmoCor apparatus (AtCor Medical Pty Ltd, West Ryde, Australia). First, short-term continuous arterial pressure waveforms were recorded by use of a hand-held tonometer (Millar, Houston, USA), simultaneously with ECG acquisition (for the synchronization of the continuous pressure waves recorded at the carotid and the femoral artery). Then, the recorded pressure waveforms were processed by proprietary software that automatically computes pulse transit time from the carotid to the femoral artery using the tangential method ^74^. Finally, cfPWV was calculated by the ratio of the distance between the two recording sites (calculated as the length from the suprasternal notch to femoral artery minus the length from the carotid artery to the suprasternal notch) to the pulse transit time. CfPWV measurements were performed with the subject at the supine position after 5 min resting period.

Noninvasive estimation of the aortic pressure waveforms was performed by the SphygmoCor System (AtCor Medical Pty Ltd), as previously described^79,80^. Radial pressure waves were first recorded by applanation tonometry and central pressure waves were derived by use of validated transfer functions^81^. Multiple recordings were performed in every subject to accomplish optimal quality control criteria (quality index: > 85%). Calibration of the recorded pulse waves was performed using the brachial systolic and diastolic BPs, which were measured by cuff sphygmomanometry. The accuracy of this apparatus has been previously evaluated by comparing the estimated aortic BPs with intra-aortic catheter-based BP measurements^79^. Furthermore, the reproducibility of this technique has been also found to be acceptable under several different conditions and populations^82^.

### Regression analysis

**Figure 8:**
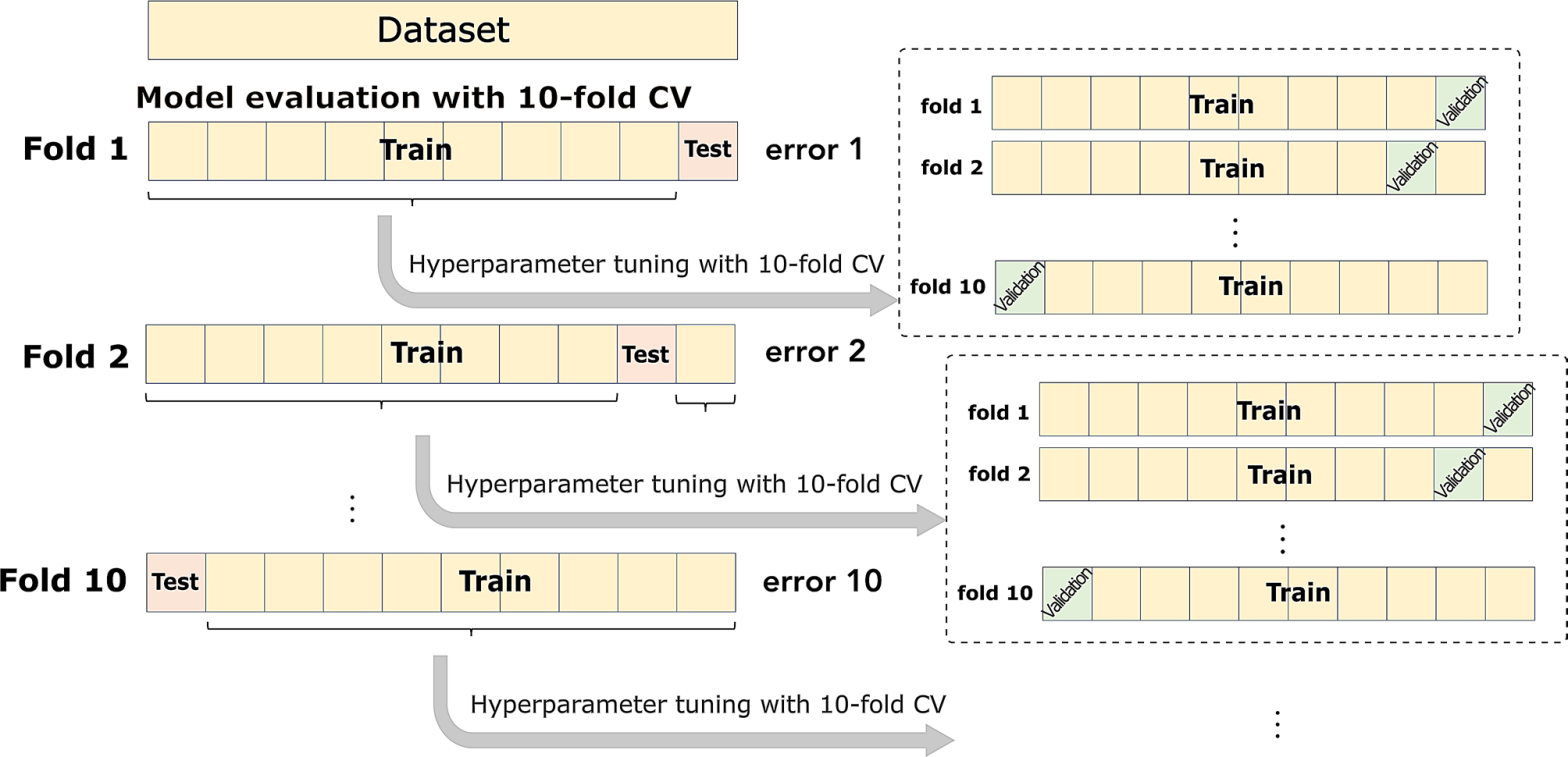
Representation of the experimental design for the evaluation of every regression model. The model evaluation of was done using tenfold cross validation (CV) (external CV). In every external fold, we performed hyperparameter tuning with tenfold CV (internal CV).

Four regression models were trained/tested to estimate the corresponding target outputs. The models that were employed were Random Forest^33^, SVR^34^, Ridge^35^, and Gradient Boosting^36^. By definition, a regression model comprises the following components: (i) the unknown hyperparameters, β, (ii) the independent variables, X_i_, and (iii) the dependent variable, Y_i_. In this analysis, the objective was to investigate whether the regression model can estimate aSBP, CO, and E_es_ from single-beat input predictors (brSBP, brDBP, HR, cfPWV, (EF)). The training/testing scheme was based on a tenfold CV scheme^83^ (Fig. 8). Concretely, all cases were divided into ten equal sets in a random manner. In each fold, one set was left out being the testing group, and the rest of sets were used as the training group to tune the parameters of the models. Hyperparameter tuning was performed internally in each fold using *GridSearch* with a tenfold CV in order to optimize the β parameters of each fold’s model (Fig. 8). The hyperparameters that were chosen to be optimized are reported in the Table 13. The hyperparameters’ values that are not reported in Table 13 were set to their default value.

**Table 13:** List of the hyperparameters which were chosen to be optimized and their corresponding values.

Model	Hyperparameters to be optimized	Values
Random forest	*max_depth*	{5, 10, 20}
	*n_estimators*	{500, 700, 1,000}
Support vector regressor	*C*	{1, 10, 100}
	*gamma*	{0.001, 0.01, 0.1, 1}
Ridge	*alpha*	{1, 10, 100, 200}
Gradient boosting	*learning_rate*	{0.01, 0.05, 1}
	*n_estimators*	{100, 500, 1,000, 1,750}

We investigated two approaches: (i) one to predict aSBP, CO, and E_es_ using brSBP, brDBP, HR, and cfPWV, and (ii) a second one to predict solely E_es_ using brSBP, brDBP, HR, cfPWV, and EF. Consequently, we evaluated the accuracy of each regression model for every target variable on a subject level. Additionally, averaging of the multiple predictions was tested as an ensemble learning approach. The training/testing pipeline was implemented using the Scikit-learn library^84^ in a Python programming environment. The pandas and numpy packages were also used^85,86^.

### In-silico validation of the model-derived predictions

We first assessed the performance of each regression model for every target variable on a subject level for the virtual population. Ten-fold CV as described above was used to evaluate the accuracy of the trained models. Moreover, we calculated the percentages of the cases whose aSBP errors met the international standards (< 5 ± 8 mmHg) of the European Society of Hypertension International Protocol^37^. The error threshold for CO was set to 0.3 and 0.5 L min^−1^ based on the objective criteria suggested by Critchley and Critchley^87^. Finally, given that the only clinically acceptable technique for measuring E_es_ is the invasive end-systolic pressure–volume relationship, there are not meta-analyses using E_es_ data. In this respect, for the E_es_ values within the range of [1, 4.5] mmHg mL^−1^, thresholds of 0.05 and 0.20 mmHg mL^−1^ should be adequate to provide an accurate estimation of E_es_.

### Sensitivity analysis for the training size

In order to assess the effect of the number of training samples on our models’ accuracy, sensitivity analysis was performed. Concretely, the regression analysis was repeated after decreasing the training size from 95 to 15% of the total number of cases. For each training size, the predictions were evaluated in terms of RMSE between the estimated and reference data. Hyperparameter tuning was implemented for each different training set under consideration.

### In-vivo validation of the model-derived aSBP predictions

**Figure 9:**
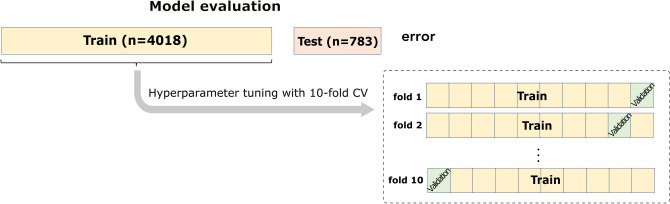
Representation of the evaluation of the synthetically trained model against in-vivo data.

Moreover, in-vivo validation was performed only for the best performing aSBP estimator, i.e., SVR. The validation was realized in two steps. First, we trained/tested an SVR model using only in-vivo data following the experimental design described in Fig. 8. Consequently, an SVR model was trained with the totality of the in-silico data (n = 4,018) and, then, was tested on the in-vivo data (n = 783), as depicted in Fig. 9. During training, hyperparameter tuning was performed using *GridSearch* with tenfold CV.

### Feature importance evaluation

We assessed the importance of each input feature using the scores returned by the Random forest model. The average importance of each feature was then calculated by averaging the scores from every fold k (k = 1, 2, … 10).

### Statistical analysis

The algorithms and the statistical analysis were implemented in Python (Python Software Foundation, Python Language Reference, version 3.6.8, Available at https://www.python.org). We performed OLS estimation of the regression coefficients using each of the target parameters, i.e., aSBP, CO, and E_es_, as dependent variable and brSBP, brDBP, cfPWV, HR, and EF (only for E_es_) as independent variables (using *statsmodels* library^88^). Hypothesis testing for each regression coefficient was realized using the *t*-stastistic. The agreement, bias and precision between the method-derived predictions and the real values were evaluated by using the Pearson’s correlation coefficient (r), the coefficient of determination (R^2^), the root mean square error (RMSE), and the normalized root mean square error (nRMSE). The computed nRMSE was based on the difference between the minimum and maximum values of the dependent variable. Bias and limits of agreement as described by^89^ were reported. The level of statistical significance was set at p < 0.05.

## Data Availability

The virtual database generated and analyzed in the present study is available from the corresponding author on a reasonable request.

## References

[1] Waddell TK, Dart AM, Medley TL, Cameron JD, Kingwell BA (2001). Carotid pressure is a better predictor of coronary artery disease severity than brachial pressure. Hypertension.

[2] Safar ME (2002). Central pulse pressure and mortality in end-stage renal disease. Hypertension.

[3] Berkenstadt H (2001). Stroke volume variation as a predictor of fluid responsiveness in patients undergoing brain surgery. Anesth. Analg..

[4] Sagawa K, Suga H, Shoukas AA, Bakalar KM (1977). End-systolic pressure/volume ratio: a new index of ventricular contractility. Am. J. Cardiol..

[5] Song-Tao A, Yan-Yan Q, Li-Xia W (2010). The severity of coronary artery disease evaluated by central systolic pressure and fractional diastolic pressure. N. Am. J. Med. Sci..

[6] Lees N, Hamilton M, Rhodes A (2009). Clinical review: goal-directed therapy in high risk surgical patients. Crit. Care.

[7] Nishimura RA, Carabello BA (2012). Hemodynamics in the cardiac catheterization laboratory of the 21st century. Circulation.

[8] Ganter MT (2016). Continuous cardiac output measurement by un-calibrated pulse wave analysis and pulmonary artery catheter in patients with septic shock. J. Clin. Monit. Comput..

[9] Hiroyuki S, Kiichi S (1974). Instantaneous pressure-volume relationships and their ratio in the excised supported canine left ventricle. Circ. Res..

[10] Suga H, Sagawa K, Shoukas AA (1973). Load independence of the instantaneous pressure-volume ratio of the canine left ventricle and effects of epinephrine and heart rate on the ratio. Circ. Res..

[11] Sagawa K (1981). The end-systolic pressure-volume relation of the ventricle: definition, modifications and clinical use. Circulation.

[12] Williams B (2018). 2018 ESC/ESH Guidelines for the management of arterial hypertension. Eur. Heart J..

[13] Papaioannou TG, Protogerou AD, Stamatelopoulos KS, Vavuranakis M, Stefanadis C (2009). Non-invasive methods and techniques for central blood pressure estimation: procedures, validation, reproducibility and limitations. Curr. Pharm. Des..

[14] Hope SA, Tay DB, Meredith IT, Cameron JD (2003). Use of arterial transfer functions for the derivation of aortic waveform characteristics. J. Hypertens..

[15] Stok WJ, Westerhof BE, Karemaker JM (2006). Changes in finger-aorta pressure transfer function during and after exercise. J. Appl. Physiol..

[16] Fetics B, Nevo E, Chen C-H, Kass DM (1999). Parametric model derivation of transfer function for noninvasive estimation of aortic pressure by radial tonometry. IEEE Trans. Biomed. Eng..

[17] Williams B (2011). Development and validation of a novel method to derive central aortic systolic pressure from the radial pressure waveform using an N-point moving average method. J. Am. Coll. Cardiol..

[18] Shih Y-T, Cheng H-M, Sung S-H, Hu W-C, Chen C-H (2014). Application of the N-point moving average method for brachial pressure waveform-derived estimation of central aortic systolic pressure. Hypertension.

[19] Udy AA, Altukroni M, Jarett P, Roberts JA, Lipman J (2012). A comparison of pulse contour wave analysis and ultrasonic cardiac output monitoring in the critically ill. Anaesth. Intensive Care.

[20] Jansen JRC (2001). A comparison of cardiac output derived from the arterial pressure wave against thermodilution in cardiac surgery patients †. Br. J. Anaesth..

[21] Langwieser N (2015). Radial artery applanation tonometry for continuous noninvasive arterial blood pressure monitoring in the cardiac intensive care unit. Clin. Res. Cardiol..

[22] Christie J (1987). Determination of stroke volume and cardiac output during exercise: comparison of two-dimensional and Doppler echocardiography, Fick oximetry, and thermodilution. Circulation.

[23] Swamy G, Mukkamala R (2008). Estimation of the aortic pressure waveform and beat-to-beat relative cardiac output changes from multiple peripheral artery pressure waveforms. IEEE Trans. Biomed. Eng..

[24] Fazeli N, Hahn J-O (2012). Estimation of cardiac output and peripheral resistance using square-wave-approximated aortic flow signal. Front. Physiol..

[25] Dabanloo, N. J., Adaei, F. & Nasrabadi, A. M. *The Performance of Neural Network in the Estimation of Cardiac Output Using Arterial Blood Pressure Waveforms*. (2011).

[26] Chen CH (2001). Noninvasive single-beat determination of left ventricular end-systolic elastance in humans. J. Am. Coll. Cardiol..

[27] Shishido P, Hayashi F, Shigemi F, Sato D, Sugimachi N, Sunagawa N (2000). Single-beat estimation of end-systolic elastance using bilinearly approximated time-varying elastance curve. Circulation.

[28] Reymond P, Merenda F, Perren F, Rüfenacht D, Stergiopulos N (2009). Validation of a one-dimensional model of the systemic arterial tree. Am. J. Physiol. Heart Circ. Physiol..

[29] Reymond P, Bohraus Y, Perren F, Lazeyras F, Stergiopulos N (2011). Validation of a patient-specific one-dimensional model of the systemic arterial tree. Am. J. Physiol. Heart Circ. Physiol..

[30] Reymond P, Westerhof N, Stergiopulos N (2012). Systolic hypertension mechanisms: effect of global and local proximal aorta stiffening on pulse pressure. Ann. Biomed. Eng..

[31] Heusinkveld MHG (2019). Augmentation index is not a proxy for wave reflection magnitude: mechanistic analysis using a computational model. J. Appl. Physiol..

[32] Avolio A (2008). Central aortic blood pressure and cardiovascular risk: a paradigm shift?. Hypertension.

[33] Liaw A, Wiener M (2002). Classification and Regression by randomForest. R News.

[34] Smola AJ, Schölkopf B (2004). A tutorial on support vector regression. Stat. Comput..

[35] Robert T (1996). Regression shrinkage and selection via the lasso. J. R. Stat. Soc. Ser. B.

[36] Natekin A, Knoll A (2013). Gradient boosting machines, a tutorial. Front. Neurorobot..

[37] O’Brien E (2010). European Society of Hypertension International Protocol revision 2010 for the validation of blood pressure measuring devices in adults. Blood Pressure Monit..

[38] Critchley LAH, Huang L, Zhang J (2014). Continuous cardiac output monitoring: what do validation studies tell us?. Curr. Anesthesiol. Rep..

[39] Nishikawa T, Dohi S (1993). Errors in the measurement of cardiac output by thermodilution. Can. J. Anaesth..

[40] Nitenberg A, Antony I, Loiseau A (1998). Left ventricular contractile performance, ventriculoarterial coupling, and left ventricular efficiency in hypertensive patients with left ventricular hypertrophy. Am. J. Hypertens..

[41] Popović Z (2000). Partial left ventriculectomy improves left ventricular end systolic elastance in patients with idiopathic dilated cardiomyopathy. Heart.

[42] Dietterich TG, Dietterich TG (2000). Ensemble Methods in Machine Learning. Multiple Classifier Systems.

[43] Joo HJ (2017). The relationship between pulse wave velocity and coronary artery stenosis and percutaneous coronary intervention: a retrospective observational study. BMC Cardiovasc. Disord..

[44] Muiesan ML (2010). Pulse wave velocity and cardiovascular risk stratification in a general population: the Vobarno study. J. Hypertens..

[45] Khoshdel AR, Carney SL, Nair BR, Gillies A (2007). Better management of cardiovascular diseases by pulse wave velocity: combining clinical practice with clinical research using evidence-based medicine. Clin. Med. Res..

[46] Monge García MI (2019). Determinants of left ventricular ejection fraction and a novel method to improve its assessment of myocardial contractility. Ann. Intensive Care.

[47] Xiao H, Qasem A, Butlin M, Avolio A (2017). Estimation of aortic systolic blood pressure from radial systolic and diastolic blood pressures alone using artificial neural networks. J. Hypertens..

[48] Ghasemi Z (2018). Estimation of cardiovascular risk predictors from non-invasively measured diametric pulse volume waveforms via multiple measurement information fusion. Sci. Rep..

[49] Bikia V (2019). Noninvasive cardiac output and central systolic pressure from cuff-pressure and pulse wave velocity: a model-based study. IEEE J. Biomed. Health Inform..

[50] Wolak A (2008). Aortic size assessment by noncontrast cardiac computed tomography: normal limits by age, gender, and body surface area. JACC Cardiovasc. Imaging.

[51] Antonini-Canterin F (2009). Arterial stiffness and ventricular stiffness: a couple of diseases or a coupling disease? A review from the cardiologist’s point of view. Eur. J. Echocardiogr..

[52] Otterstad JE (2002). Measuring left ventricular volume and ejection fraction with the biplane Simpson’s method. Heart.

[53] Simpson, R. *et al.* Comparing echocardiography and cardiac magnetic resonance measures of ejection fraction: implications for HFMRF research. In *British Cardiovascular Imaging Meeting 2018* A3.1-A3 (BMJ Publishing Group Ltd and British Cardiovascular Society, 2018). 10.1136/heartjnl-2018-BCVI.6.

[54] Reant P (2010). Systolic time intervals as simple echocardiographic parameters of left ventricular systolic performance: correlation with ejection fraction and longitudinal two-dimensional strain. Eur. J. Echocardiogr..

[55] Howard JP (2019). Artificial intelligence for aortic pressure waveform analysis during coronary angiography. JACC.

[56] Huttunen JMJ, Kärkkäinen L, Lindholm H (2019). Pulse transit time estimation of aortic pulse wave velocity and blood pressure using machine learning and simulated training data. PLoS Comput. Biol..

[57] Stergiopulos N, Westerhof BE, Westerhof N (1998). Physical basis of pressure transfer from periphery to aorta: a model-based study. Am. J. Physiol.-Heart Circ. Physiol..

[58] Trachet B (2010). Numerical validation of a new method to assess aortic pulse wave velocity from a single recording of a brachial artery waveform with an occluding cuff. Ann. Biomed. Eng..

[59] Papaioannou TG, Vardoulis O, Stergiopulos N (2012). The, “systolic volume balance” method for the noninvasive estimation of cardiac output based on pressure wave analysis. Am. J. Physiol.-Heart Circ. Physiol..

[60] Huttunen JMJ, Kärkkäinen L, Honkala M, Lindholm H (2020). Deep learning for prediction of cardiac indices from photoplethysmographic waveform: a virtual database approach. Int. J. Numer. Methods Biomed. Eng..

[61] Shi Y, Lawford P, Hose R (2011). Review of zero-D and 1-D models of blood flow in the cardiovascular system. BioMed. Eng. OnLine.

[62] Willemet M, Vennin S, Alastruey J (2016). Computational assessment of hemodynamics-based diagnostic tools using a database of virtual subjects: application to three case studies. J. Biomech..

[63] Vennin S (2017). Identifying hemodynamic determinants of pulse pressure: a combined numerical and physiological approach. Hypertension.

[64] Westerhof N, Lankhaar J-W, Westerhof BE (2009). The arterial Windkessel. Med. Biol. Eng. Comput..

[65] Chen C-H (1998). Coupled systolic-ventricular and vascular stiffening with age. J. Am. Coll. Cardiol..

[66] Pak PH, Maughan WL, Baughman KL, Kieval RS, Kass DA (1998). Mechanism of acute mechanical benefit From VDD pacing in hypertrophied heart: similarity of responses in hypertrophic cardiomyopathy and hypertensive heart disease. Circulation.

[67] Feldman MD (1996). Acute cardiovascular effects of OPC-18790 in patients with congestive heart failure: time- and dose-dependence analysis based on pressure-volume relations. Circulation.

[68] Senzaki H, Chen C-H, Kass DA (1996). Single-beat estimation of end-systolic pressure-volume relation in humans: a new method with the potential for noninvasive application. Circulation.

[69] Starling MR (1987). The relationship of various measures of end-systole to left ventricular maximum time-varying elastance in man. Circulation.

[70] Devereux RB (2012). Normal limits in relation to age, body size and gender of two-dimensional echocardiographic aortic root dimensions in persons ≥15 years of age. Am. J. Cardiol..

[71] Lu Z, Mukkamala R (2006). Continuous cardiac output monitoring in humans by invasive and noninvasive peripheral blood pressure waveform analysis. J. Appl. Physiol..

[72] Langewouters GJ (1982). Visco-elasticity of the Human Aorta in Vitro in Relation to Pressure and Age.

[73] Segers P (2008). Three- and four-element Windkessel models: assessment of their fitting performance in a large cohort of healthy middle-aged individuals. Proc. Inst. Mech. Eng..

[74] Vardoulis O, Papaioannou TG, Stergiopulos N (2013). Validation of a novel and existing algorithms for the estimation of pulse transit time: advancing the accuracy in pulse wave velocity measurement. Am. J. Physiol.-Heart Circ. Physiol..

[75] Liu J (2017). Patient-specific oscillometric blood pressure measurement: validation for accuracy and repeatability. IEEE J. Transl. Eng. Health Med..

[76] Papaioannou TG (2019). The influence of resting heart rate on pulse wave velocity measurement is mediated by blood pressure and depends on aortic stiffness levels: insights from the Corinthia study. Physiol. Meas..

[77] Tousoulis D (2020). Acute exposure to diesel affects inflammation and vascular function. Eur. J. Prev. Cardiol..

[78] Papaioannou TG (2019). Arterial stiffness and subclinical aortic damage of reclassified subjects as stage 1 hypertension according to the new 2017 ACC/AHA blood pressure guidelines. VASA.

[79] Papaioannou TG (2016). Accuracy of commercial devices and methods for noninvasive estimation of aortic systolic blood pressure a systematic review and meta-analysis of invasive validation studies. J. Hypertens..

[80] Ioakeimidis N (2020). Acute effect of heat-not-burn versus standard cigarette smoking on arterial stiffness and wave reflections in young smokers. Eur. J. Prev. Cardiol..

[81] Karamanoglu M, O’Rourke MF, Avolio AP, Kelly RP (1993). An analysis of the relationship between central aortic and peripheral upper limb pressure waves in man. Eur. Heart J..

[82] Siebenhofer A, Kemp C, Sutton A, Williams B (1999). The reproducibility of central aortic blood pressure measurements in healthy subjects using applanation tonometry and sphygmocardiography. J. Hum. Hypertens..

[83] Wong T-T (2015). Performance evaluation of classification algorithms by k-fold and leave-one-out cross validation. Pattern Recogn..

[84] Pedregosa F (2011). Scikit-learn: Machine Learning in Python. JMLR.

[85] McKinney, W. Data Structures for Statistical Computing in Python. In *Proceedings of the 9th Python in Science Conference* 51–56 (2010). 10.25080/Majora-92bf1922-00a.

[86] Oliphant TE (2006). A guide to NumPy.

[87] Critchley LAH, Critchley JAJH (1999). A meta-analysis of studies using bias and precision statistics to compare cardiac output measurement techniques. J. Clin. Monit. Comput..

[88] Seabold, S. & Perktold, J. *statsmodels: Econometric and statistical modeling with python*. (2010).

[89] Bland JM, Altman DG (1986). Statistical methods for assessing agreement between two methods of clinical measurement. Lancet.

